# Identifying the Degree of Gene Reuse During Repeated Adaptation

**DOI:** 10.1111/mec.70180

**Published:** 2025-11-21

**Authors:** Samridhi Chaturvedi, Lakshmi Vineesha Digumarthi, Garima Setia

**Affiliations:** ^1^ Department of Ecology & Evolutionary Biology Tulane University New Orleans Louisiana USA

**Keywords:** adaptive evolution, ecological genomics, gene reuse, genomics methods, parallel adaptation, probability, quantification, repeated adaptation

## Abstract

The degree or extent of gene reuse during repeated adaptation offers key insights into the genomic constraints on evolution. Although many studies have identified signs of genomic repeatability, a thorough synthesis of methods for detecting gene reuse and estimating its extent is missing. In this review, we first propose a simple framework for studies aimed at identifying gene reuse during repeated adaptation using genomic data. Next, we examine existing approaches to (i) detect gene set overlap, perform significance testing, and perform multivariate dimensionality reduction, (ii) distinguish between gene and allele reuse while emphasising methods for detecting allele reuse, (iii) explore models to identify the mechanisms behind repeated adaptation, and (iv) address issues and potential solutions to differentiate true gene reuse from methodological artifacts. Our review highlights standardised methods developed using genomic data to identify gene reuse. We also note that, although few studies quantify allele reuse, conducting such analyses is essential because it adds a more detailed layer to predicting evolutionary paths. Finally, several strategies can be used to cross‐validate signals of gene reuse. These should be applied to confirm true positives, as biological and methodological artifacts can bias predictions. By synthesizing current methods and outlining a robust analytical framework, we provide a roadmap for enhancing the accuracy and reliability of gene reuse detection in adaptive evolution.

## 1 Background

**FIGURE 1:**
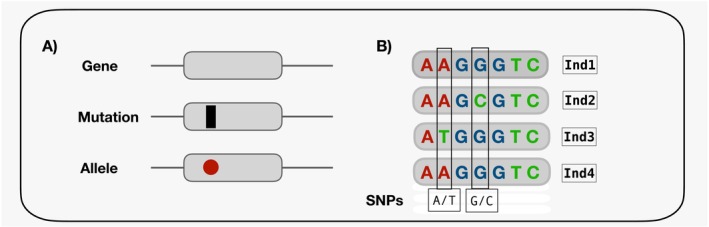
Examples of genomic features used in genome scans to identify candidate genes for repeated adaptation. (A) Representation of a gene, mutation, and allele. Grey boxes indicate genes positioned along a continuous genome sequence (black line). A black bar within a gene denotes a point mutation, and the red dot represents an allele that could result from this mutation. (B) Example of nucleotide sequences for a single gene in four individuals (Ind1–Ind4). Variations in nucleotide bases among individuals indicate single nucleotide polymorphisms (SNPs); here, two SNPs are shown: A → T and G → C.

**FIGURE 2:**
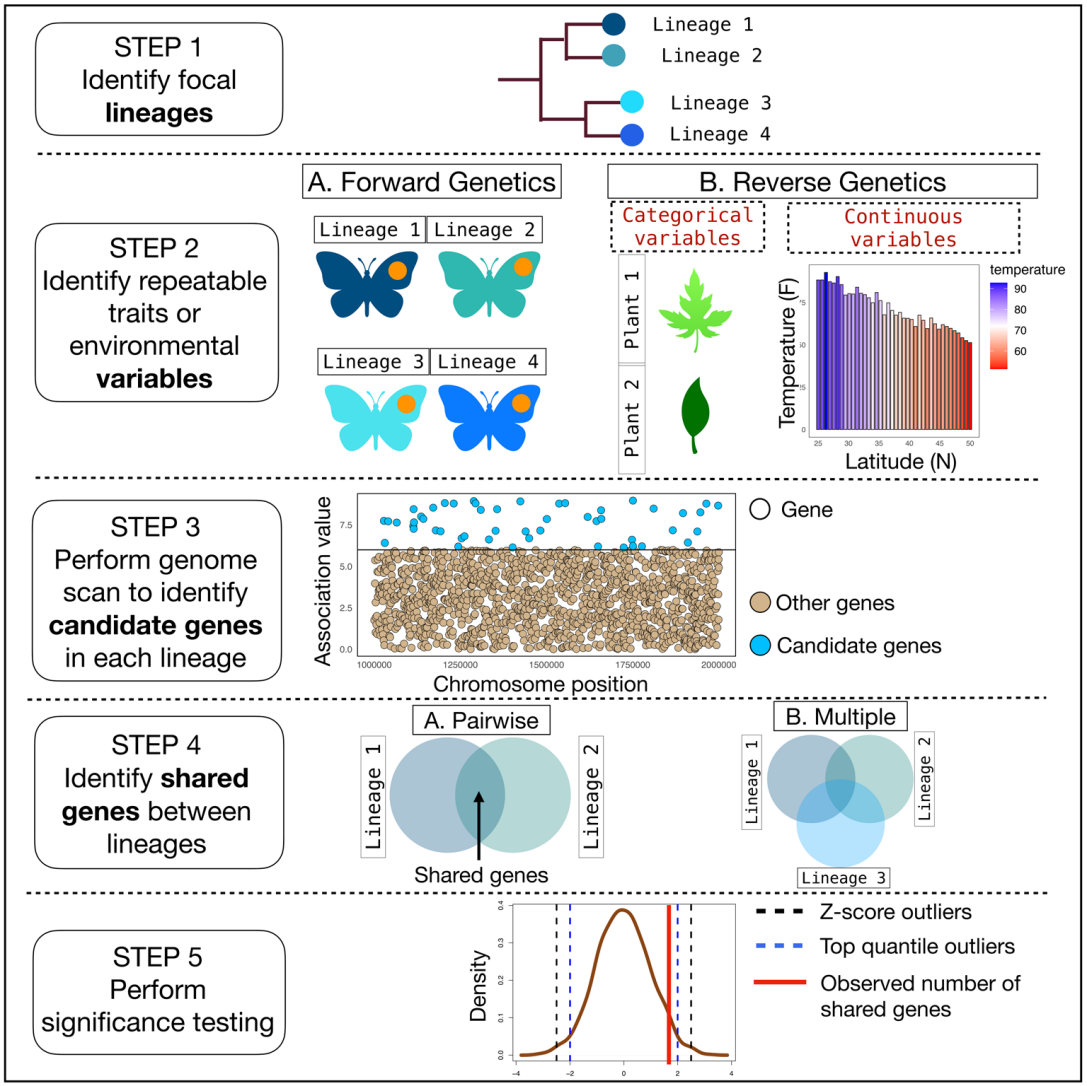
Conceptual framework for identifying genomic gene associated with repeated adaptation. This figure presents a stepwise framework for detecting and validating genomic regions involved in repeated adaptation. Step 1: Define comparative groups and identify focal lineages. Step 2: Determine relevant traits, such as wing spot pattern, that have repeatedly evolved in each lineage under similar selective pressures (e.g., predation or environmental variation). Traits may be categorical (e.g., host plant species) or continuous (e.g., precipitation, temperature). When traits are known, forward genetics can be used to identify the genomic basis of adaptation; when unknown, reverse genetics can be applied (see main text). Step 3: Use genome scan approaches to identify genes with exceptional association to the trait or environment (“candidate genes”). In the example Manhattan plot, each point is a gene, with candidates (blue) exceeding an association threshold (black line) and other genes in brown. Step 4: Identify candidate genes shared across independent lineages using gene set overlap methods, ranking by top quantiles or Z‐scores to build a robust dataset for repeated adaptation tests. Step 5: Evaluate whether observed gene reuse exceeds random expectations using parametric tests. The figure also highlights common approaches for detecting candidate genes, including genome‐wide association tests, normal distribution–based outlier detection, and cross‐lineage overlap assessments.

In nature, independent lineages (see Box 1, Glossary) frequently evolve similar traits in response to comparable selective pressures—a phenomenon known as repeated adaptation (see Box 1, Glossary; Arendt and Reznick 2008; Conte et al. 2012; Martin and Orgogozo 2013; Cerca 2023; Bohutínská and Peichel 2024). Repeated adaptation emphasises the predictability of evolution at both phenotypic and genetic levels (Martin and Orgogozo 2013). At the molecular scale, this repeatability can manifest through shared changes in the mutations, alleles, and regulatory pathways that underlie adaptation (Box 1, Figure 1; Cerca 2023). Thus, identifying repeated adaptation at the molecular level begins with recognising instances of gene reuse, which arise when independent lineages utilise the same genes (Figure 2), potentially conferring fitness advantages in similar contexts and environments (Bohutínská and Peichel 2024).

BOX 1 **Glossary.**

**Mutation:** A permanent change in the DNA sequence. In this review, we focus mainly on point mutations, where a single nucleotide changes at a specific site in the genome.

**Allele:** One of two or more variants of a gene present at a specific location in the DNA sequence. Multiple alleles can occur within the same gene.

**Single Nucleotide Polymorphism (SNP):** A site in the genome where a single base differs among individuals.

**Gene:** A segment of DNA that codes for a heritable trait. In this review, “gene” refers broadly to any unit of analysis in genome scans, though in practice such scans are applied to SNPs and can also target non‐coding regions of the genome.

**Genome Scans:** A genome‐wide approach, similar to genome‐wide association studies (GWAS), that tests many genes across the genome to identify regions significantly associated with a particular trait (trait‐GWAS) or environmental variable (environmental‐GWAS). Exceptionally high statistical association values suggest a potential role in adaptation, and such genes are often referred to as “adaptive genes.” The output is typically a genome‐wide distribution of *p*‐values or test statistics for each gene or variant.

**Gene Reuse:** When the same genes, though not necessarily the same mutations or alleles, contribute to repeated adaptation.

**Allele Reuse:** When the same allele of a gene contributes to repeated adaptation, often due to allele sharing between lineages.

**Lineage:** A genetically distinct unit that can include populations, species or even kingdoms, essentially sharing a common ancestor at some point in time in the past.

**Repeated Adaptation:** The independent evolution of similar traits in different lineages in response to similar environmental pressures, leading to improved fitness in each lineage. This may involve the same or different alleles, genes, or traits, and can arise through gene or allele reuse.

While significant progress has been made in understanding the likelihood of gene reuse (Rosenblum et al. 2014), the factors influencing the degree of gene reuse—or the extent of genomic repeatability—remain poorly understood. Clarifying these factors is crucial for unraveling how genomic constraints affect evolutionary predictability (Speed and Arbuckle 2017; Yeaman et al. 2018; Pearless and Freed 2024). Furthermore, evidence of repeated adaptation comes primarily from studies focused on traits that are driven by simple genetic architectures, such as single genes of large effect. Polygenic traits are more complex to study, as their genetic architecture may involve the reuse of several genes with minor effects, which are harder to detect using traditional genomic sequencing approaches. Additionally, while there are numerous documented cases of repeated adaptation due to gene reuse, it is not always clear whether allele sharing also contributes to repeated adaptation. Evidently, multiple knowledge gaps remain in our understanding of gene reuse.

**FIGURE 3:**
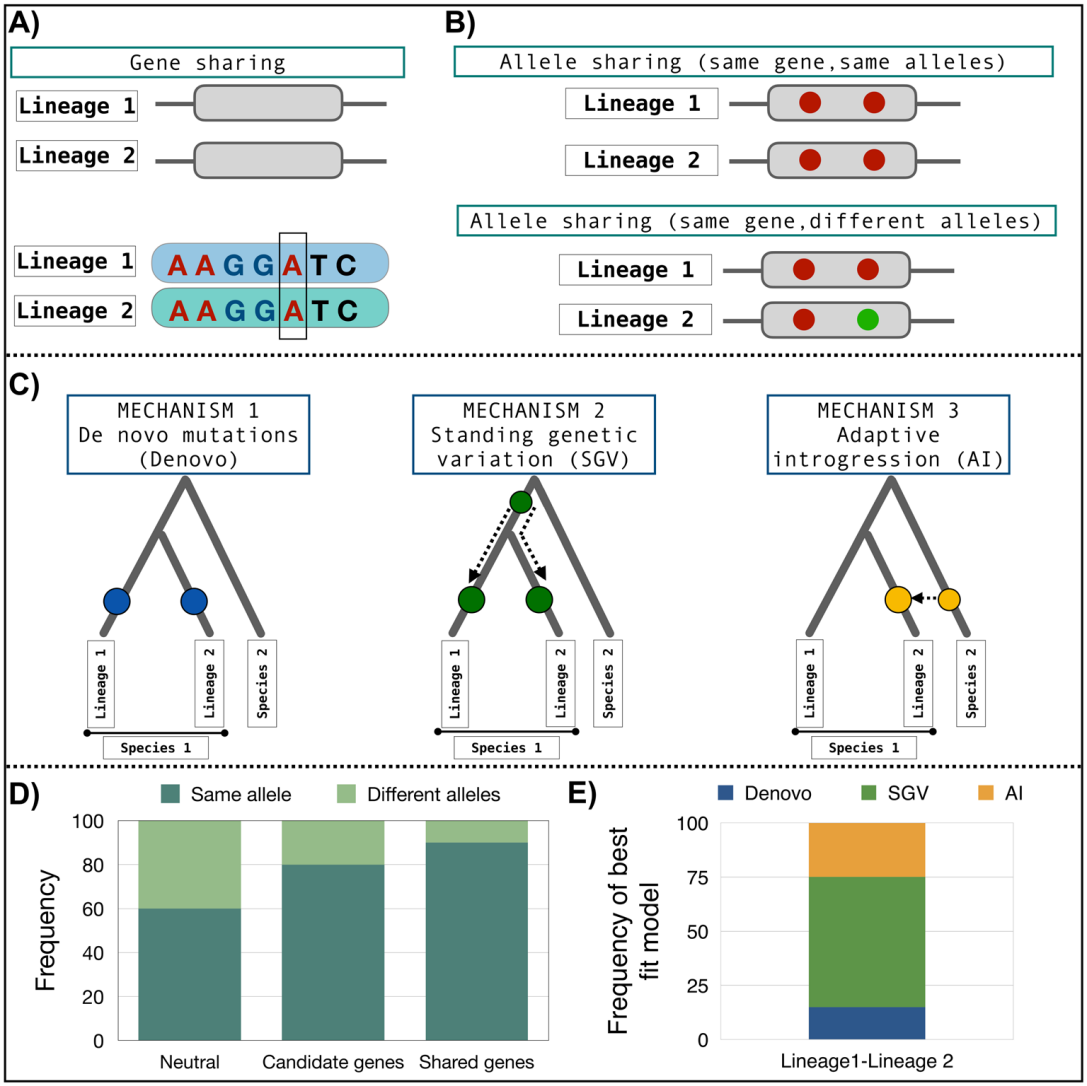
Quantifying gene reuse and allele sharing across lineages. The figure presents an analytical framework for assessing gene reuse, allele sharing, and repeated adaptation modes. (A) Gene reuse can occur through shared genes or alleles. Allele sharing could occur through the sharing of the same allele on the same gene in two or more lineages. Here, each gene is represented as a grey box positioned along a continuous genome sequence represented by black lines (similar to Figure 1 above). We assume that the same gene is shared between Lineage 1 and Lineage 2. The second panel represents a gene sequence in which a SNP is shared between the two lineages. This is to depict how SNPs are identified in genome scan approaches. (B) We can also test for allele reuse between lineages. Each filled circle indicates an allele for a (biallelic) SNP on the gene. In the case of the same gene, same allele, all alleles are identical between lineages (all are red circles). In the case of the same gene, different alleles, one of the alleles differs, indicated here as a green circle instead of the red circle. (C) Gene or allele reuse can arise through different evolutionary mechanisms, including Mechanism 1: De novo mutations: Two mutations denoted as blue circles occur independently in each lineage of the same species, Mechanism 2: Standing genetic variation (SGV), an ancestral mutation denoted as green circle is inherited in both lineages of the same species, and Mechanism 3: Adaptive introgression (AI): A beneficial mutation denoted as an golden yellow circle is transferred from species 2 to lineage 2 of species 1. (D) The extent of allele reuse for each queried gene is quantified by measuring the frequency of the same alleles or different alleles for neutral outliers, selected outliers, and shared outliers between lineages (modified from figure 3 in (Zhang et al. 2019)). (E) The likelihood of each mode is quantified for each shared gene or allele, showing the relative contribution of de novo mutations, SGV, and AI in driving repeated adaptation. This framework allows for a systematic evaluation of molecular parallelism and the evolutionary mechanisms underlying genomic repeatability.

Identifying the genomic bases of adaptive evolution has shown that repeated adaptation can occur through different mechanisms that introduce or maintain adaptive genes in multiple lineages that are evolving in response to similar selective pressures. Three main mechanisms can cause repeated adaptation: independent mutations or de novo mutations (two or more lineages independently acquire the same beneficial mutation, leading to the same trait), standing genetic variation (SGV) (a mutation that existed in the ancestral population at low frequency before divergence is later favored in multiple lineages facing similar selective pressures), and migration (a beneficial mutation arises in one population and then spreads to others through gene flow; Arendt and Reznick 2008; Lee and Coop 2017; Figure 3C). When evidence of repeated adaptation through gene reuse is identified, it is not always clear which mechanism was involved, requiring further research into the genomic basis of evolutionary repeatability (Lee and Coop 2017; Lai et al. 2024).

Knowledge gaps surrounding gene reuse exist for two main reasons: (1) past studies lacked genome‐wide sequencing data, thereby limiting our ability to detect the nature of genetic changes underlying repeated adaptation, and (2) there was a lack of suitable models capable of integrating background population structure (genetic structuring due to random drift), the genomic architecture (number and effect of genes, linkage disequilibrium) and genetic interactions (pleiotropy or epistasis) into inferences of adaptation and, consequently, the probability and extent of repeatability. However, recent advances in genomic sequencing and method development have transformed studies of repeated adaptation. The decreasing cost of high‐throughput sequencing has made it feasible to generate data at higher depth and broader genomic coverage in natural, non‐model organisms, enabling more confident detection of repeatable, genome‐wide signatures of evolution. Simultaneously, new computational models that use genomic data and account for genomic architecture and genetic interactions provide increasingly robust inferences about gene reuse. These innovations open new avenues for disentangling the mechanisms driving repeated adaptation.

Building on recent methodological advances, we provide a timely review of approaches for identifying gene reuse in repeated adaptation and quantifying the extent of repeatability. We focus on genomic constraints that limit the extent of gene reuse and complicate its identification. We then compare existing methods, evaluate their strengths and limitations, and propose strategies to improve molecular repeatability detection while minimising false positives. Our review centers on five key areas: (1) Identifying genomic constraints that influence the detection of gene reuse in repeated adaptation, (2) Summarising and comparing current methods for quantifying gene reuse, (3) Distinguishing gene reuse from allele reuse and evaluating methods to quantify allele reuse, (4) Identifying models that infer the mechanism of repeated adaptation through gene or allele reuse, and (5) Identifying problems and solutions to validate signals of gene reuse. Additionally, we provide a simple framework that can be used to quantify the extent of gene reuse during adaptation. By synthesising these insights, we aim to provide a practical guide for researchers, enhancing the accuracy and reliability of detecting genomic repeatability in adaptive evolution.

## 2 Genomic Constraints on the Degree of Gene Reuse in Repeated Adaptation

The probability and degree (or extent) of gene reuse are related but distinct aspects of repeatability, both shaped by evolutionary and genomic factors. The probability of gene reuse refers to the likelihood that the same genes will be involved in adaptive responses across independent lineages, influenced by the net fitness effects of mutations (Conte et al. 2012; Stern 2013). It is expected that genes harboring beneficial mutations that increase fitness in one lineage will have a higher probability of being reused in other lineages, as they will be readily available for selection as standing genetic variation or through gene flow. The degree of gene reuse reflects the extent to which the same genes are reused across lineages during adaptation. These two concepts are linked: a higher probability of gene reuse increases the expected degree of gene reuse across lineages. Several factors can act as “genomic constraints” and affect both the probability and the degree of gene reuse. Primary factors include time since divergence, gene flow, maintenance of standing genetic variation, and genetic architecture of traits. We discuss each of these briefly below (but see also relevant reviews on this topic as cited).

Divergence time between lineages is a key determinant of the likelihood and degree of gene reuse, with gene reuse decreasing as divergence increases (Bohutínská et al. 2020; Chaturvedi et al. 2022). Gene reuse is more likely in taxa that share a common ancestor with similar genetic architectures than in distantly related groups (discussed and reviewed in detail in Conte et al. 2012; Bohutínská and Peichel 2024). Shared ancestral variation and conserved pathways could be important factors (among others) driving gene reuse in closely related species.

Standing genetic variation (SGV) increases the likelihood of gene reuse in natural populations, as beneficial alleles present in the gene pool can facilitate repeated adaptation (Elmer and Meyer 2011; Conte et al. 2012; Ralph and Coop 2015; Hoban et al. 2016; Bomblies and Peichel 2022). This is supported by modeling studies and examples from empirical research. For instance, shared selective sweeps following relatively recent selection pressures can play an essential role in repeated evolution (Lee and Coop 2017), and the probability of repeated genetic evolution from SGV increases with stronger selection and larger effective population sizes, particularly for genes with significant phenotypic effects (MacPherson and Nuismer 2017).

High gene flow can increase the likelihood and the extent of gene reuse by helping beneficial alleles spread across populations. For instance, the “transporter” hypothesis suggests that in stickleback populations, freshwater‐adapted alleles are transferred via hybridization and recombination, becoming part of the standing genetic variation in marine populations. When marine sticklebacks colonize new freshwater habitats, natural selection can reassemble the adaptive genotype, resulting in repeated phenotypic adaptation across geographically distant populations (Schluter and Conte 2009). Low gene flow among structured populations does not preclude the development of the same trait through independent *denovo* mutations, although this can be a less common driver of repeated adaptation (Bohutínská et al. 2020; Chaturvedi et al. 2022).

Genetic architecture of traits can influence the likelihood and degree of gene reuse, especially for quantitative traits. When studying the genetic architecture of traits, understanding the effect size of a gene is essential because small‐effect genes have a lower and more subtle impact on traits whereas large‐effect genes have significant influence on an individual's traits. When conducting genome scans, small‐effect genes may be rare (rare variants), and large‐effect genes are often more common (common variants). The genetic architectures of quantitative traits can include many small‐effect genes (polygenic), a single large‐effect locus (monogenic), a few large‐effect genes (oligogenic), multiple large‐effect loci, or a mix of these (Yeaman 2022). The number of small or large‐effect genes available in SGV affects the probability of repeated adaptation. When comparing phenotypic and genetic repeatability, a highly polygenic architecture—where many small‐effect alleles contribute to a trait—can lower genetic repeatability because of redundancy, meaning that multiple genetic combinations can produce the same phenotype. Conversely, when only a few large‐effect alleles segregate at appreciable frequencies, reduced redundancy constrains the number of genetic routes to adaptation, leading to faster and more repeatable evolutionary outcomes (Whiting, Paris, Parsons, et al. 2022).

The four factors mentioned above have been reviewed and discussed in detail in previous reviews (see Conte et al. 2012 (SGV, gene flow, genetic architecture); Rosenblum et al. 2014 (SGV, gene flow); Cerca 2023 (genetic architecture); Bohutínská and Peichel 2024 (divergence times)). Thus, we move beyond these factors and focus here on three factors that can complicate the detection of gene reuse: linkage disequilibrium (non‐random association of alleles at linked genes), pleiotropy (a single gene contributes to multiple phenotypic traits), and epistasis (interaction between genes that masks the effect of one gene) (Hartl 2020). Natural selection can favour specific combinations of alleles that create and sustain linkage disequilibrium (hereafter referred to as LD). This can influence the rate and pattern of recombination and promote the inheritance of adaptive haplotypes, thereby increasing gene reuse. Pleiotropy determines whether mutations are beneficial across multiple traits, limiting or promoting reuse based on migration‐selection balance and fitness trade‐offs (Otto 2004). Epistasis, through interactions between genes, influences whether exactly the same genetic solutions arise in different populations or whether alternative genetic pathways are favoured. Thus, these factors influence the genetic architecture of traits and impact gene reuse, influencing the probability and extent of repeated adaptation (Sanjuán and Elena 2006). They shape how selection, recombination, and genetic interactions determine the reuse of adaptive genes across populations. Below, we examine how these factors structure patterns of genome‐wide signatures of repeatability during adaptation, as they can complicate reliable detection of gene reuse.

### 2.1 Linkage Disequilibrium

LD plays a central role in shaping both the probability and degree of gene reuse by influencing how alleles are inherited together. Strong LD, often caused by low recombination rates, chromosomal inversions, or supergenes, maintains beneficial haplotypes across populations, increasing the reuse of specific genes (Yeaman 2013; Tigano and Friesen 2016). For example, chromosomal inversions can affect patterns of LD across the genome and are also involved in repeated adaptation. Chromosomal inversions prevent the breakup of favourable combinations of alleles by suppressing recombination within the inverted region. This leads to increased LD among loci within the inversion, maintaining these co‐adapted gene complexes in the face of gene flow. The LD generated within an inversion can extend across large segments of the chromosome. Inversions contribute disproportionately to repeated adaptation by linking multiple locally adaptive alleles, strengthening selection on the associated supergene and potentially speeding up the adaptive process (Hoffmann and Rieseberg 2008). In sunflowers (*Helianthus* spp.), for example, genomic regions with inversions in one species often show strong genome‐wide association signals (with focal phenotypes and environment) in other species lacking the inversion, and inversions overall have disproportionate contributions to repeated divergences (Soudi et al. 2023; Huang et al. 2025). This suggests that while local adaptation is flexible—relying on different genes across species—it can also involve a repeatable genomic basis, with structural variants such as inversions playing a key role in driving adaptive evolution (Soudi et al. 2023). Another pattern of LD involves polygenic adaptive traits, among which tight LD can lead to the repeated selection of the same genomic regions, increasing the degree of gene reuse. However, in genomic regions with high recombination, selection on polygenic traits may favour different combinations of genes across lineages, thereby reducing the degree of gene reuse (Orr and Coyne 1992; Yeaman 2022). Thus, strong LD promotes gene reuse, while weak LD and high recombination diversify adaptive responses and could lead to lower gene reuse, particularly in polygenic traits (Barghi et al. 2020).

Methodologically, LD can complicate the detection of shared adaptive genes and potentially lead to false positives while masking true signals, particularly among physically proximate genes and can reduce the resolution of genome scans (Figure 2, Box 1, Glossary) (Terwilliger 2001; Teo et al. 2009; Wang et al. 2010; Bailey‐Wilson and Wilson 2011; Chaturvedi et al. 2022). When using a genome scan approach to identify candidate genes (those which show significantly high association with a trait or environmental variable, Figure 2, Step 3) and infer gene reuse, it is essential to control for LD (or apparent LD), which can be inflated by neutral population structure (due to random processes such as genetic drift, gene flow or mutations) and demographic history. Window‐based analyses can help account for the non‐independence of linked markers and control for random variation associated with genes (or variants) (Booker et al. 2024). Briefly, a window‐based method in genome scans involves splitting the genome into fixed‐size segments known as “windows,” and examining genetic variation or association signals within each segment (Hoban et al. 2016). LD‐aware genome–environment association (GEA) methods, or mixed models that incorporate kinship matrices (Weir et al. 2006), also explicitly account for background LD and population structure. Together, these approaches improve the reliability of outlier detection by reducing false positives due to correlated allele frequencies and help isolate genes that are more likely to be repeatedly involved in adaptation across populations.

### 2.2 Pleiotropy

Pleiotropy influences gene reuse by modulating the fitness consequences of mutations across multiple traits (Stern 2013; Lai et al. 2024). The Fisher‐Orr model predicts that genes with low pleiotropy are more likely to be reused, as mutations in these genes have minimal deleterious trade‐offs (Fisher 1999; Orr 2005). Additionally, large‐effect genes may have fewer pleiotropic constraints and can be particularly advantageous in two scenarios: (1) early in the adaptation process, when big steps are most beneficial, they provide large phenotypic shifts that move populations quickly toward a new fitness optimum, and (2) under migration–selection balance, their strong selective advantage allows them to persist despite the influx of maladaptive alleles from migration, whereas small‐effect alleles are more easily swamped by gene flow (Otto 2004). For example, the melanocortin 1 receptor (Mc1r) gene is a classic case of repeatability across deep evolutionary time, influencing adaptive colour polymorphism in diverse vertebrates from fish to mammoths (Manceau et al. 2010), and its widespread reuse may be partly due to minimal interactions with other genes, allowing easier modification with less pleiotropic disruption (Mundy 2005; Whiting et al. 2024).

Genes with low pleiotropic constraint are more likely to be reused during adaptation because their effects on multiple traits are less conflicting, allowing adaptive variants to persist within populations (Lotterhos et al. 2018; Auge et al. 2019). Pleiotropic constraint can depend on the form of pleiotropy. Pleiotropy can take different forms: synergistic pleiotropy occurs when a single gene improves multiple traits, whereas antagonistic pleiotropy has beneficial effects on one trait but harmful effects on others, creating fitness trade‐offs (McGee et al. 2016). Synergistic pleiotropy can facilitate rapid, coordinated changes across traits, promoting coadaptation, and such loci may be maintained as standing genetic variation that supports repeated adaptation to similar environments (Rennison and Peichel 2022). Conversely, antagonistic pleiotropy can impose constraints on adaptation through fitness trade‐offs, thereby reducing gene reuse over time (Zajitschek and Connallon 2018; Barghi et al. 2020; Lai et al. 2024). Finally, the extent to which pleiotropy promotes, or limits gene reuse depends on the modularity of gene function and the structure of the selective landscape.

Methodologically, pleiotropy can make it difficult to interpret signals of repeated adaptation. A pleiotropic gene may appear as an outlier in genome‐wide scans, but its association with adaptation might actually reflect effects on an unmeasured or unknown trait. In other words, a gene reused across populations could influence multiple traits—some studied, some not—making it challenging to determine which trait is the true target of selection. Integrating phenotype data can therefore help clarify the specific trait(s) driving the observed genomic signal (Chebib and Guillaume 2021). Furthermore, because pleiotropy causes a single gene to affect multiple traits, and linkage causes nearby genes to show correlated signals, it can be difficult to tell whether a repeated signal reflects true functional reuse of the same gene or just linkage to another selected locus (Tyler et al. 2016; Chebib and Guillaume 2021). Multi‐trait analyses with integration of phenotypic data, genetic correlation studies, fine‐mapping techniques, and specialised statistical methods are crucial for identifying pleiotropic genes, with methods such as PLEIO providing frameworks for analysing shared genes across multiple diseases and traits while accounting for genetic correlations and heritability (Lee et al. 2021).

### 2.3 Epistasis

Epistasis—defined as the interactions between alleles at different genes—plays a crucial role in determining the likelihood and extent of gene reuse by altering the fitness impacts of mutations in a way that is dependent on the interacting genes. Strong epistasis can sometimes promote alternative genetic pathways, which may decrease gene reuse among populations (Blanquart et al. 2013). When epistatic interactions consistently favour specific genetic changes across various populations, they can reinforce patterns of repeated evolution. A notable example of this is seen in Baltic cod populations, where positive synergistic epistasis drives repeated adaptation in response to rising salinity levels due to climate change (Stern et al. 2022). Consequently, epistasis can either facilitate or limit gene reuse, depending on whether it steers adaptation toward shared solutions or leads to alternative, lineage‐specific evolutionary pathways (Blanquart et al. 2013; Huang et al. 2014).

Like pleiotropy, epistasis complicates the detection of shared genetic effects by distorting estimates of trait measures, hiding individual gene effects, and increasing analytical complexity, which can lead to misinterpretation of results (false negatives and false positives) by making it harder to identify true signatures of gene reuse due to interactive influences (Ni et al. 2020; Batista et al. 2024). Thus, researchers must be aware of the existence of potential epistatic interactions in their genomic data and factor in this possibility when performing genome scans and interpreting signals of gene reuse during instances of repeated adaptation. Furthermore, while LD, pleiotropy, and epistasis can independently influence inferences of gene reuse, they can also interact with each other, which can further affect the genomic architecture of adaptation and influence genome scans. In the following sections, we further discuss these issues when implementing specific methods and designing studies on repeated adaptation.

## 3 A Simple Framework to Quantify Gene Reuse

Before we discuss the current methods used to identify gene reuse and quantify its extent during repeated adaptation, we outline a general study design, which consists of five main steps (summarised in Figure 1).

Step 1 involves identifying focal lineages (Box 1, Glossary) that have independently adapted to similar environmental conditions or selective pressures. These lineages may represent populations within a species or closely related species.

Step 2 focuses on characterising the traits or environmental variables that may underlie repeated evolution. In Figure 2, Lineages 1–4 represent butterfly lineages that have each evolved an orange warning spot in response to avian predation, a classic case of repeated evolution under selective pressure for aposematism (Set 1). Two approaches have been used to identify the genomic basis of adaptation. A forward genetics approach begins with identifying an adaptive phenotype and then determining the genes responsible for it, often using approaches such as quantitative trait locus (QTL) mapping, recombinant inbred line mapping, or genome‐wide association studies (GWAS) (Bomblies and Peichel 2022) (Figure 2, Step 2A). Alternatively, the reverse genetics approach starts by identifying genes that show signatures of selection, typically through genome scans, and then testing the phenotypic effects of these genes through functional studies (Stapley et al. 2010; Bomblies and Peichel 2022; Figure 2, Step 2B). Environmental variables can be either categorical (e.g., herbivore colonises a novel host plant repeatedly by utilising the same genes; Figure 2, Step 2B) or continuous (e.g., temperature gradients across latitudes, where lineages distributed along this gradient independently evolve similar thermal tolerances; Figure 2, Step 2B). These environmental or ecological variables serve as predictors in subsequent genotype‐by‐environment analyses (GEA).

Step 3 involves performing genome scans (Box 1, Glossary) independently within each lineage to identify trait‐associated genes (Trait GWAS) or environment‐associated genes (Environmental GWAS). We call the genes that are outliers in this analysis “candidate genes”. These genes are expected to be involved in adaptation and can also be referred to as “adaptive genes”. The other genes are neutral or non‐significantly associated with the trait or environment. Two main methods can be employed for conducting genome scans. First, genotype‐by‐environment association (GEA) approaches detect genes significantly associated with environmental or phenotypic variables, implicating them as adaptive. Second, *F_ST_*‐based outlier detection methods identify genes showing unusually high genetic differentiation between populations, indicative of divergent selection and possible candidate genes that might show independent evolution between lineages.

Step 4 quantifies the overlap in candidate genes across lineages (set of genes per lineage) to detect gene reuse. This is typically done using gene set intersection or overlap methods (see methods section below, e.g., Venn diagrams), and can include pairwise comparisons (Figure 2, Step 4A) or analysis involving multiple lineages (Figure 2, Step 4B). The genes shared across lineages are referred to as “shared genes”.

Step 5 involves formal significance testing to assess whether the observed number of shared genes exceeds expectations under a null model of no reuse. This typically involves generating a null distribution by randomising sets of outlier genes, then comparing the observed overlap to this distribution. Common approaches include Z‐score–based tests or top‐quantile enrichment tests (Figure 2, Step 5). A randomization test can then be performed to ask if the observed number of shared genes between lineages is significantly higher than expected under a null model. If the observed overlap significantly exceeds expectations, it suggests that gene reuse during repeated adaptation is more frequent than expected by chance.

This five‐step framework provides a structured and replicable approach to investigating gene reuse and testing hypotheses about the repeatability of the genetic basis of adaptation across lineages. While this approach is standard, the impact of the underlying genetic architecture and gene interactions (as implied by genes associated with a trait or environment) on detecting gene reuse has been largely overlooked until recently. Below, we provide an overview of the methods currently used to quantify gene reuse, describe their applications, and discuss their advantages and limitations. We also provide a summary of these methods in Table 1.

**TABLE 1:** Summary of key methods and statistical tests used to quantify gene reuse in repeated adaptation studies. The table describes each metric, the index it produces, its interpretation, key citations, advantages, disadvantages, consideration of genetic architecture (LD) and genetic interactions, and available implementations in R or Python. Methods are divided into four categories as described in the main text: Gene set overlap methods, significance testing methods, methods tested using genomic data, and multidimensionality reduction methods. Custom implementations are required for some specialized methods.

Methods	Index produced	Interpretation	Citations	Implementation in R/Python	Advantages	Disadvantages	Genetic architecture consideration
*Gene set overlap*							
Jaccard Similarity Index	Jaccard Index (0 to 1)	Measures proportion of shared genes between sets. Higher values indicate substantial gene reuse.	Bailey et al. (2015)	R: vegan::vegdist(method = “jaccard”), Python: sklearn.metrics.jaccard_score	Simple and scalable	Highly sensitive to the number of gene included in the dataset (Salvatore et al. 2020)	Does not account for LD, pleiotropy, or epistasis
Cluster Separation Score (CSS)	CSS	Measures how well adaptive gene clusters separate between populations. High scores indicate potential reuse.	Jones et al. (2012)	[Custom implementation needed]	Accounts for correlation in high‐dimensional data	Lacks a formal framework for significance testing	Can incorporate correlations due to LD
*Significance testing*							
Pairwise Hypergeometric Test	*p*	Tests if two gene sets overlap more than expected by chance. A low *p*‐value indicates significant gene reuse.	Plaisier et al. (2010), Cheng et al. (2021)	R: phyper(), Python: scipy.stats.hypergeom	Widely used for gene set overlap testing	May overestimate gene set overlap due to assumption of gene independence, which is often violated in co‐expressed or functionally related genes	Does not account for LD, pleiotropy, or epistasis
Fisher's Exact Test	*p*	Tests for significant enrichment of gene overlap between sets.	Fisher (1922)	R: fisher. test(), Python: scipy.stats.fisher_exact	Robust to noise in input data	Not scalable to large sample sizes	Limited insight into specific genetic architecture
FDR Correction	Adjusted *p* (*q*‐value)	Controls for multiple testing when analysing gene set overlap.	Benjamini and Hochberg (1995)	R: p.adjust(method = “fdr”), Python: statsmodels.stats.multitest.multipletests(method = “fdr_bh”)	Captures shared genetic architecture in multiple populations	Can confound effects of hierarchical gene ontology structure	Indirectly reflects shared genetic basis
Spearman's Rho	Correlation coefficient (−1 to 1), *p*	Measures rank correlation between gene properties in different conditions.	Spearman (2010)	R: cor.test(method = “spearman”), Python: scipy.stats.spearmanr	Provides genome‐wide perspective	Limited to detecting non‐linear relationships only	May reflect linked selection under certain models
Cochran–Mantel–Haenszel (CMH) Test	*p*	Evaluates association between gene presence and adaptation across multiple strata.	Mantel and Haenszel (1959), Barghi et al. (2019)	R: mantelhaen.test(), Python: statsmodels.stats.contingency_tables. StratifiedTable	Enables detection of multivariate parallelism	Assumes data homogeneity and is not suitable for pool‐seq data (Wiberg et al. 2017)	Captures pleiotropy if multivariate traits are modelled
Wilcoxon Rank Sum Test	*p*	Compares ranked distributions of gene properties across groups. Low *p*‐values suggest systematic reuse of gene classes.	Wilcoxon (1945)	R: wilcox. test(), Python: scipy.stats.ranksums	Useful for enrichment analysis across datasets	Biased when small gene sets are tested against much larger complementary sets (Fang et al. 2012)	May partially capture LD depending on implementation
X‐fold enrichment	*p*	Compares ranked distributions of gene properties across groups. Low *p*‐values suggest systematic reuse of gene classes.	Chaturvedi et al. (2018), (2023)	Custom implementation in R	Useful for enrichment analysis across datasets	Affected by LD	May partially capture LD depending on implementation
*Significance testing using genomic data*							
SuperExactTest	*p*, odds ratio	Evaluates significance of overlaps among multiple gene sets. Useful for multi‐species parallel adaptation studies.	Wang et al. (2015)	R: SuperExactTest package	Supports multi‐set intersections, including shared genes across multiple sets	Can confound biological organisation with genetic architecture and inflate overlap estimates (Lawhorn et al. 2018)	Does not account for LD, pleiotropy
Zhang & Kumar's Test for Convergent Evolution	*p*, convergence score	Tests whether amino acid changes occur more often than expected under neutrality.	Zhang and Kumar (1997)	[Custom implementation needed]	Flexible for different evolutionary hypotheses	Excludes informative sites that do not converge to the exact same amino acid, potentially missing gene interaction effects (Rey et al. 2018)	Assumes gene act independently unless modelled otherwise
Null‐W Method	*p*	Tests for excess overlap beyond what is expected under a null model of independent evolution.	Yeaman et al. (2016)	[Custom implementation needed]	Designed to detect constrained evolution	Requires simulations to generate null expectations	Can account for LD if modelled in the null and integrated with genomic features
C‐scores and *P_a,lik_*	C‐score, likelihood ratio (*P_a,lik_*)	Quantifies adaptive convergence at specific sites.	Yeaman et al. (2018)	[Custom implementation needed]	Allows for comparison across multiple taxa	Sensitive to genome scan thresholds and computationally intensive	Can incorporate pleiotropy if designed appropriately
PicMin	*p*, similarity score	Identifies parallel genetic changes and tests for significant reuse, particularly in phylogenetic contexts.	Booker et al. (2020)	https://github.com/TBooker/PicMin	Highlights convergent signals across studies	Sensitive to genome scan thresholds and computationally intensive	No explicit modelling of LD or epistasis
*Multivariate methods*							
AFVapeR	Eigenvalues	Divides the genome into windows of an equivalent number of SNPs, and within each window performs eigen decomposition over normalised allele frequency change vectors (AFVs), each derived from a replicated pair of populations/species.	Whiting, Paris, Parsons, et al. (2022)	R: afvaper()	Can identify direction of parallel allele frequency change and incorporate different sampling designs	Susceptible to sampling design of common ancestor and phylogenetic relatedness	Considers LD and recombination but not genetic interactions
EigenTensor	Covariates	Identifies whether the same genetic variants or pathways are repeatedly used.	De Lisle et al. (2024)	PCA‐based methods (e.g., singular value decomposition in R/Python), tensor decomposition packages (e.g., NumPy, TensorFlow in Python), or specialised evolutionary genetics tools (e.g., evolqg in R for quantitative genetics)	Supports visualisation of complex results	Reduces complex genomic variation into few dimensions, which may be sensitive to noise and miss nuanced signals	Depends on the granularity of input data

## 4 Current Methods to Quantify Gene Reuse During Repeated Adaptation

Several statistical approaches have been used to identify gene reuse during repeated adaptations and inform conclusions about the extent of gene reuse. When assessing gene reuse, the standard methods for identifying the number of reused genes between lineages rely on set overlap techniques and significance testing to determine if the number of reused genes is significant compared to a null model. A few recent methods have now been explicitly standardised using genomic data. Additionally, some methods treat genomic data on a multivariate level to identify gene reuse from the repeatability of allele frequency change. We highlight these methods below by classifying them into four categories: (1) gene set overlap methods, (2) significance testing methods, (3) methods standardised using genomic data, and (4) multivariate dimensionality reduction methods. We want to emphasise that although many methods in the third and fourth categories are based on standard statistical tests, their limitations and strengths have been identified through testing with genomic data to detect gene reuse explicitly.

### 4.1 Gene Set Overlap Methods to Identify Reused Genes

A common approach for quantifying gene reuse during repeated adaptation is the gene‐set overlap method, which identifies genes that are shared between two or more sets of data (Bohutínská and Peichel 2024). In mathematical terms, this corresponds to finding the intersection of sets by determining which elements are common across the sets being compared. In biomedical genomics studies, this concept is widely applied to assess the extent of overlap in candidate genes between different groups. For example, gene‐set overlap methods are used to test whether the same genes are differentially expressed under two treatments, or whether the same genetic variants are associated with a trait (e.g., height or disease) in different lineages. These methods are often visualised using Venn diagrams (Figure 2, Step 4) and have been increasingly employed in evolutionary genomics to test for gene reuse during repeated adaptation (Figure 2, Step 3). It is expected that overlapping or shared genes between two sets are of adaptive interest.

The Jaccard similarity index, initially developed in ecology, is a simple yet informative measure for quantifying the degree of similarity between two sets. It is calculated by dividing the number of shared elements (intersection) by the total number of unique elements (union) across sets. In the context of gene reuse, it is used to estimate the proportion of shared genes between lineages relative to the total number of candidate genes identified across both lineages (Salvatore et al. 2020). Clustering methods, such as the Cluster Separation Score (CSS) and Differentiation Index (DI), group genes based on similarity in selection signals or allele frequencies and evaluate the distinctness of those clusters. These methods have been applied in studies of repeated adaptation to test whether clusters of candidate genes in different lineages show consistent signals of adaptation through exceptional association with the environment or trait being tested (Jones et al. 2012; Morales et al. 2019). While conceptually straightforward, useful for summarising overlap, and valuable tools when sufficient genomic and phenotypic data are available, the methods described above do not independently assess statistical significance. They can be affected by study design factors such as sample size, the number of individuals per lineage, and the number of candidate genes identified in selection scans. To address these issues, below we highlight some alternative approaches that can effectively perform significance testing.

### 4.2 Significance Tests to Identify Statistically Meaningful Genes

To assess whether the observed overlap is greater than that expected by chance, the gene‐set overlap methods are typically coupled with significance testing, using either parametric or non‐parametric methods, to compare observed overlap to a null distribution (Figure 2, Step 5). Non‐parametric methods offer a flexible alternative for significance testing in gene reuse studies, particularly when assumptions of normality or independence are violated. These methods are well suited for incorporating complex genomic features such as polygenic architecture or linkage. In the sections that follow, we summarise commonly used statistical approaches for significance testing, and provide a concise overview of their implementation, assumptions, advantages, and limitations—including considerations related to linkage disequilibrium (LD) and genetic architecture (see Table 1).

1. Hypergeometric test: This test is a commonly used method in gene reuse studies. It evaluates whether the observed number of shared outlier genes between two lineages is greater than would be expected by chance, assuming random sampling without replacement from a finite gene set. This test has been frequently applied in genome‐wide selection scans to assess adaptive gene overlap between populations (Plaisier et al. 2010; Cheng et al. 2021). Although widely accessible and easy to implement, a key limitation is its assumption of independence among genes. This assumption is often violated due to LD and background population structure, potentially inflating false positives.
2. Fisher's exact test: This test provides a similar statistical framework to the hypergeometric test but is particularly suited for smaller sample sizes. It evaluates whether there is a statistically significant association between two categorical variables—in this case, gene identity and reuse status—by computing the probability of observing a given distribution under the assumption of independence. This method has been applied in gene reuse studies to determine whether shared genes occur more frequently than expected under random distribution (Abatangelo et al. 2009; Holliday et al. 2016). While more robust than the hypergeometric test for small datasets, Fisher's exact test can become computationally intensive in large‐scale genomic analyses, limiting its scalability.
3. Spearman's rank correlation coefficient (*ρ*) is a non‐parametric statistic that assesses the strength and direction of a monotonic relationship between two ranked variables. In gene reuse studies, it can be used to test for genome‐wide correlation in selection scores (e.g., *p*‐values or effect sizes) between lineages (Arciero et al. 2018). This approach helps identify shared genes but may fail to capture non‐linear associations or interactions among genes. Additionally, it can be confounded by background population structure, especially when allele frequencies correlate with environmental gradients for reasons unrelated to adaptation (Coop et al. 2010).
4. The Cochran–Mantel–Haenszel (CMH) test is a stratified test used to detect consistent allele frequency differences across multiple comparisons, accounting for stratification by lineage or replicate. It has been adapted in gene reuse studies to identify genes showing repeated allele frequency changes across populations or lineages (Orozco‐Terwengel et al. 2012; Barghi et al. 2020). This test is beneficial for detecting repeatability at the allele level under binary selection regimes, such as case–control comparisons; however, it is not well‐suited for detecting gene reuse in response to continuous environmental variables (Huh et al. 2015; Figure 2, Step 2).
5. The Wilcoxon signed‐rank test, also referred to as the Mann–Whitney *U* test, is a non‐parametric method used to compare differences between two groups without assuming a normal distribution. In the context of gene reuse, this test has been used to assess whether the distributions of selection scores for reused genes differ significantly from those of non‐reused genes (Thorhölludottir et al. 2025). It is useful when working with small sample sizes or non‐normally distributed data. However, like other non‐parametric methods, it may have limited statistical power, particularly when working with polygenic traits.
6. *X‐fold enrichment*: This method quantifies the degree to which the number of shared genes observed between lineages exceeds a null expectation, typically expressed as a fold‐change. It has been applied in empirical studies of Melissa blue butterflies and Timema stick insects to assess whether repeated adaptation involves the same genes more often than expected by chance (Chaturvedi et al. 2018, 2022). While intuitive and straightforward to calculate, the accuracy of X‐fold Enrichment depends on pairwise or multiple lineage comparisons with the latter being more reliable.

Although widely used in studies of repeated adaptation, the methods described above are standard statistical models used to perform simple significance tests by considering a null model. They are limited in their use, as they were not designed to identify adaptive genes or quantify gene reuse, and they lack standardisation with genomic data. Consequently, these models can be affected by sample size, genetic drift, genomic architecture, background population structure, and the non‐random distribution of adaptive genes across the genome. As with all genome‐wide analyses, applying multiple testing correction—such as the false discovery rate (FDR)—is essential to control for false positives. While FDR correction is effective in reducing Type I errors, it may be overly conservative in some cases, potentially masking true adaptive signals. However, recent advancements in the field of evolutionary genomics have resulted in the standardisation of models using genomic data and studying repeated adaptation, potentially leading to more accurate estimates of gene reuse. We describe these methods in the next section.

### 4.3 Methods Standardised Using Genomic Data

In this section, we highlight methods that have been developed to identify shared genes between lineages and quantify the extent of gene reuse using genomic data. We want to caution readers that each of these methods requires genome scans (Box 1, Glossary) for identifying candidate genes and shared genes between lineages (Figure 2). The power of the genome scans is essential here. A powerful genome scan that can detect small‐effect genes requires good sample sizes (multiple individuals sequenced per lineage) and a sequencing depth that enables reliable genotype likelihood calculations (see Section 7 for a more detailed discussion). Lastly, polygenic architectures can make it challenging to detect small‐effect variants underlying repeatable traits. Therefore, a powerful genome scan will improve the performance of each of these methods. Below, we identify five methods that have been standardised using genomic data and have been applied to identify signatures of repeated adaptation.

1. The SuperExactTest, implemented in an R package developed by Wang et al. (2015), is specifically designed to assess significance in the overlap of multiple gene sets. It generalises the exact test to situations involving more than two sets and calculates the likelihood that observed intersections among gene sets occur by chance. In studies of repeated adaptation, this method is particularly useful when comparing gene reuse across three or more lineages. SuperExactTest is scalable for large datasets and provides an efficient means to evaluate complex reuse patterns. However, its statistical assumptions are similar to those of other overlap‐based tests described above, and it does not inherently account for LD or gene interactions.
2. Zhang and Kumar's test for repeated evolution is a specialised method used primarily to detect molecular convergence at the amino acid sequence level. It evaluates whether the same amino acid changes have independently evolved across lineages more often than expected by chance, offering strong support for repeated evolution at the sequence level (Zhang et al. 2019). This method has been informative for protein‐coding genes but is less applicable to non‐coding regulatory regions or structural variation, both of which may also play crucial roles in repeated adaptation. Thus, its utility in studies of gene reuse depends on the genomic features under investigation.
3. The Null‐W method is a specialised approach designed to detect gene reuse across lineages while accounting for linkage disequilibrium (Yeaman et al. 2016). It compares the average squared correlation coefficient (*ρ* ^2^) between top candidate adaptive genes and a background set of genes to assess whether shared genes show stronger signals of selection than expected. This method was originally applied in studies of lodgepole pine and interior spruce (Yeaman et al. 2016) and is particularly useful for studies involving highly structured genomes. However, this method requires adequate sample sizes per lineage and may lack statistical power in small datasets, limiting its general applicability.
4. The C‐score index is a statistical framework for quantifying constraints on repeated adaptation by testing whether observed gene reuse exceeds expectations under a null model of independent evolution (Yeaman et al. 2018). Its main advantage for measuring gene reuse is its ability to distinguish between two types of constraints that shape repeated adaptation: genotype–trait redundancy (GT‐redundancy) and genotype–fitness redundancy (GF‐redundancy). GT‐redundancy refers to the number of genetic pathways that can produce the same phenotypic trait value. Low GT‐redundancy means only a few genes or specific allele combinations can generate that trait, while high GT‐redundancy means many interchangeable genetic routes exist. In high GT‐redundancy systems—such as those assumed in classical quantitative genetics or omnigenic models—independent adaptation events are more likely to use different genetic solutions, lowering repeatability. In contrast, low GT‐redundancy limits mutational options and increases the likelihood of repeatedly targeting the same genes. GF‐redundancy captures variation in fitness among genotypes that yield the same phenotype. Even if multiple genetic routes can produce the trait (high GT‐redundancy), only some genotypes may be optimal for fitness due to pleiotropic effects, epistasis, allele effect size differences, or linkage architecture. Low GF‐redundancy means only a narrow subset of genotypes are both phenotypically correct and selectively advantageous, increasing repeatability; high GF‐redundancy allows many equally fit solutions, reducing repeatability.
  The C‐score index distinguishes between GT and GF‐ redundancy by calculating three indices: *C hyper*, *C chisq*, and *P_a,lik_*. The index *C hyper*, derived from a hypergeometric test, evaluates whether the number of shared genes across lineages is greater than expected by chance and can be applied to both binary and continuous trait data, making it most suitable for pairwise lineage comparisons. *C chisq*, based on a chi‐square framework, tests for deviations from expected reuse frequencies across any number of lineages but can only be calculated for continuous data. Higher C‐scores indicate stronger constraints on adaptation, suggesting that the same genes are repeatedly used across evolutionary events. Finally, *P_a,lik_*, an adaptive likelihood score, estimates the probability that a given gene contributes to adaptation, with higher values indicating weaker constraints and greater flexibility in adaptive responses. Applying this method to genomic data involves identifying adaptive gene through genome scans (e.g., *F_ST_*, XP‐CLR, or dN/dS, Figure 2, Step 3), identifying shared genes across lineages, generating null expectations using a hypergeometric framework, and computing C‐scores and *P_a,lik_* to assess significance.
  This modelling framework explicitly incorporates genetic architecture and genetic interactions to determine constraints on the diversity of genetic routes to adaptation. Genetic architecture is represented by parameters defining the number of genes affecting a trait, their effect sizes, and the mutational target size—summarised as the effective adaptive target (EAT). These values depend on GWAS‐based identification of genomic regions associated with the trait and the effect‐size estimates for each gene. Pleiotropic interactions are modelled by allowing individual genes to influence multiple traits, with mutational effects represented as vectors in multivariate trait space. This structure introduces trade‐offs, as mutations beneficial for one trait may harm others, lowering their likelihood of contributing to adaptation. Such antagonistic pleiotropic effects constrain the gene accessible to selection, thus shaping the EAT. When the model incorporates migration in this framework, it is possible that this antagonistic pleiotropy can interact with gene flow and reduce repeatability (Battlay et al. 2024). By explicitly simulating interactions between pleiotropy and genetic architecture, the framework provides a mechanistic basis for predicting when and why evolution follows predictable, repeated genetic paths. Although computationally intensive for large datasets, it offers a rigorous approach for testing gene reuse and comparing adaptive constraints across systems.
5. PicMin is a statistical method designed to study the contribution of individual genes to repeated adaptation across lineages (Booker et al. 2023). This method is similar to the parametric and non‐parametric statistics mentioned above, with the advantage that it has been standardised explicitly for genomic data to estimate the likelihood that observed adaptive changes at a particular gene exceed expectations under neutral evolution. Implementing PicMin with genomic data involves identifying candidate genes via genome scans (such as *F_ST_* outliers, selection gradients, or dN/dS ratios), and finding shared genes across lineages (Figure 2, Step 2–4) before applying the PicMin framework to implement significance testing. The shared gene set is the input data that PicMin uses for its statistical analyses.
  PicMin uses the set of shared genes and ranks each gene relative to all genes to generate a rank score and assign a *p*‐value to evaluate statistical significance. This approach is not limited to pairwise comparisons and can be expanded to multiple lineages to measure gene reuse (pairwise versus more than two, Figure 2 Step 4), increasing statistical power as more lineages show adaptive signals. A key advantage of PicMin is its flexibility in handling various evolutionary scenarios and its ability to reduce false positives compared to simpler overlap‐based metrics. The model's limitations include not explicitly accounting for genomic architecture and linkage effects.
  The effectiveness of this method depends on the power of the genome scans and the number of lineages analysed; specifically, it performs much better with four or more lineages. It can detect adaptive genes even with low‐power genome scans involving four or more lineages, including small‐effect genes that may underlie repeated adaptation. However, with fewer than four lineages, the number of genes detected is also low, and the method can miss small‐effect genes or rare genes. Despite these limitations, PicMin offers a robust statistical approach for detecting repeated molecular evolution and inferring gene reuse in adaptive evolution.

While the methods described above provide a strong framework for detecting and quantifying gene reuse, they do not capture whether adaptation involves the same alleles across populations or if allele frequencies shift in the same direction (increase or decrease) over time. Allele frequency shifts in the same alleles over time can be repeatable between lineages when each lineage is exposed to similar environmental pressures. Repeatable allele frequency shifts can indicate evolutionary paths and help predict the direction of evolution, showing whether independently evolving lineages are likely to converge on similar trajectories or diverge over time. Below, we summarise methods that can assist in making these predictions when studying repeated adaptation.

### 4.4 Multivariate Dimensionality Reduction Methods to Detect Shared Allele Frequency Changes

Repeated adaptation at the genomic level can be observed through allele reuse or allele sorting, where adaptive haplotypes are shared either through inheritance as common variation (SGV) or via gene flow, and are independently selected across lineages (Booker et al. 2023). When common haplotypes are reused, it leads to repeated changes in allele frequency across multiple genes within the haplotype, creating a strong signal of consistent multivariate trajectories in allele frequency space (Whiting, Paris, Van Der Zee, and Fraser 2022). Therefore, to identify and measure repeatability, one can examine allele reuse or the direction of allele frequency shifts in shared alleles between lineages. Below, we outline two methods that offer different perspectives on identifying gene reuse by analysing allele frequency changes. Although these methods utilise genomic data, they do not explicitly consider genomic architecture or genetic interactions. Nonetheless, they provide a powerful means of detecting repeatable evolutionary patterns through their unique application.

1. Eigentensor‐based methods provide a robust framework for detecting gene reuse by analysing patterns of genetic covariance across populations undergoing repeated adaptation (De Lisle et al. 2024). This approach involves constructing a genomic covariance matrix from allele frequency data, gene expression profiles, or other genomic features across independent evolutionary events, and then performing eigen decomposition to extract eigentensors—multidimensional axes that capture dominant patterns of shared genetic variation. In this context, eigentensors are analogous to principal component vectors but applied to the decomposition of covariance tensors, such as the P matrix, which describes phenotypic covariance among traits. Eigentensors quantify how P matrices diverge among populations or environments, with such divergence influencing both the capacity and direction of future evolution. Each eigentensor represents an independent axis of variation, and the leading eigentensor and its first eigenvector pinpoint the trait combinations showing the most significant shifts in covariance structure. Examining divergence along these eigentensor‐defined axes reveals whether evolutionary change aligns with prevailing selection pressures, offering insight into the predictability of adaptation. Comparisons between observed P matrix divergence and theoretical predictions for microevolutionary change provide further tests of predictability; for example, De Lisle et al. (2024) showed that theoretical models explained over 30% of divergence across the lake–stream habitat boundary in stickleback, suggesting a substantial degree of evolutionary repeatability. If multiple populations adapt along the same eigentensor, it implies a repeatable genetic basis for adaptation via gene reuse.
  Implementing this approach in genomic datasets involves computing the covariance structure of allele frequencies for candidate alleles (SNP based allele frequencies, see Box 1, Glossary for definition of SNP), followed by tensor decomposition techniques such as principal component analysis (PCA), singular value decomposition (SVD), or higher‐order tensor factorization using tools like NumPy (Python) or evolqg (R). The resulting eigenvalues and eigentensors identify the directionality of allele frequency change, allowing researchers to assess the repeatability of adaptation. Advantages of eigentensor‐based methods include their ability to condense complex genomic variation into a small number of interpretable dimensions and their robustness to noise, as they emphasise major trends during repeated adaptation.
  Although eigentensor‐based methods were initially developed to analyse divergence in phenotypic (P) covariance matrices, their application to genomic data represents an extension of this framework to the genetic (G) level. The approach assumes that patterns of allele frequency covariance across populations can approximate the underlying structure of genetic variance and covariance. This connection is conceptually justified because selection acts on phenotypic variance, which in turn reflects the genetic covariance structure driving evolutionary responses (Cheverud 1988; De Lisle et al. 2024). However, when applied to genomic datasets, the method does not explicitly separate genetic effects from environmentally induced (co)variance, and thus may conflate adaptive genetic divergence with environmentally driven phenotypic plasticity. Moreover, P matrices can often display more meaningful dimensions of variation than G matrices due to the inclusion of environmental effects. Consequently, eigentensor‐based methods are most effective for identifying repeated adaptation when traits have relatively simple genetic architectures or when genomic data can be linked to phenotypic traits through forward‐genetic approaches (Figure 2, Step 2).
2. AF‐vapeR (Allele Frequency Vector Analysis of Parallel Evolutionary Responses) is a method inspired by the Eigen‐tensor method described above (Whiting, Paris, Van Der Zee, and Fraser 2022). AF‐vapeR is a multivariate genome‐scan approach that constructs, within each SNP window (see SNP definition in Box 1, Glossary), an allele‐frequency change (AFV) matrix composed of per‐SNP allele frequency changes across multiple population pairs. Eigen decomposition of this AFV matrix yields eigenvalues and eigenvectors that reflect the structure of allele‐frequency changes in multivariate space. A dominant first eigenvalue (and its corresponding eigenvector) indicates a shared direction of allele‐frequency change across populations, thereby providing evidence of complete repeatability (i.e., the same allele increasing in frequency in all comparisons). Peaks on the first eigenvector flag those genomic windows where repeated divergence occurs. If a second eigenvector also shows a significant eigenvalue. In that case, this signals a second axis of repeated change—indicating “multiparallelism”, which arises when different mutations at the same gene are favoured in various lineages.
  AF‐vapeR does not require prior identification of candidate genes/alleles. This method uses SNP‐based allele frequencies and a window‐based genome scan approach to calculate eigenvectors per window. Within each window, per‐SNP allele frequency change is calculated between population pairs, defined to represent directional change (e.g., ecotype, phenotype, habitat, or timepoint A → B) under designs standard to repeated adaptation studies, such as unique replicate pairs, standard ancestor designs, or multi‐comparisons with replacement. Unlike clustering‐based approaches such as CSS or CMH, which detect only full “parallelism” or repeatability under assumptions of low within‐cluster and high between‐cluster differentiation, AF‐vapeR can detect multiple axes of repeatability in both clustered and unclustered scenarios, using a permutation‐based null that accommodates different sampling designs. While highly repeatable allele frequency change is often interpreted as evidence for repeated, independent selection, AF‐vapeR does not explicitly test for selection, and other processes (such as drift or LD) can also generate these patterns of repeatability. The method works with any sequencing data, but whole‐genome data performs better. Significant limitations include sensitivity to sampling design, filtering of SNPs during variant calling based on minor allele frequency (e.g., MAF filtering threshold of ≥ 5%, though 1% may suffice), and genome scan window sizes. Smaller windows increase eigenvalue variance, whereas larger windows may capture linked SNPs reflecting haplotype‐level allele reuse; thus, linkage decay estimates can guide parameter choice, and multi‐scale scans can help filter spurious outliers. Interpretation of empirical *p*‐values from the permuted null requires caution—low *p*‐values identify the strongest relative signals. Still, enrichment of low *p*‐values genome‐wide indicates more gene reuse than expected. AF‐vapeR has successfully recovered known and novel candidate genes across empirical datasets, highlighting its utility for characterising repeatable evolutionary processes. AF‐vapeR can also distinguish between mechanisms of repeated adaptation, which we describe below.

Overall, each of the methods described above represents a significant step forward in identifying gene reuse during repeated adaptation. They offer different approaches for identifying adaptive genes and calculating the degree of gene reuse. The degree of reuse may also differ depending on the underlying mechanism of repeated adaptation. Tools like AF‐vapeR and others (described below) can assist in uncovering these mechanisms, adding further depth to studies of repeated adaptation.

## 5 Distinguishing Gene Versus Allele Reuse and Quantifying Allele Reuse

Distinguishing between gene reuse and allele reuse is critical for understanding repeated adaptation, yet these concepts are often conflated, leading to potential misinterpretations of evolutionary patterns (Rosenblum et al. 2014). Gene reuse refers to the repeated involvement of the same gene in adaptation across populations or species, regardless of whether the underlying alleles differ (Figure 3A; gene sharing). In contrast, allele reuse occurs when the same allele(s) at a specific gene are independently selected across different populations or species (Figure 3B; allele sharing).

To accurately infer allele reuse, it is essential to consider whether the shared alleles are identical‐by‐state (IBS)—having the same nucleotide sequence—or identical‐by‐descent (IBD)—inherited from a common ancestor (Powell et al. 2010; Henden et al. 2018). IBD alleles, which are necessarily IBS, often reflect shared genetic ancestry and are more likely to be present at appreciable frequencies in related populations, thereby increasing the likelihood of reuse during adaptation. In contrast, IBS alleles that are not IBD may represent independent de novo mutations that arose in different lineages in response to similar selective pressures. The presence of such independently originated but functionally equivalent alleles points to strong evolutionary constraints, but could lead to a lower degree of repeatability at the molecular level due to genetic redundancy.

Synonymous substitutions—those that do not change the amino acid sequence—result in functionally identical alleles. If two individuals possess the same newly derived synonymous allele, these alleles would be considered IBS at that locus. Conversely, nonsynonymous substitutions may lead to functionally divergent alleles, potentially reducing allele reuse even if the same gene is reused. Thus, identifying whether gene reuse involves synonymous or nonsynonymous changes is central to understanding the extent and nature of allele reuse.

With this background, it becomes evident that allele reuse can also be the primary mechanism underlying the genetic repeatability of adaptation, particularly among closely related lineages or in systems with ongoing gene flow (McCulloch et al. 2021). However, gene reuse can also occur without allele reuse. For example, different alleles at the same gene may contribute to adaptation in different lineages, indicating repeatability at the genic but not allelic level. To empirically test this, one can compare the frequency of candidate genes in focal lineage pairs that share both the same gene and the same allele versus those that share the same gene but harbor different alleles (Figure 3A; Wang et al. 2021). If the same alleles are reused, we expect stronger molecular repeatability; if different alleles underlie adaptation at the same gene, it suggests that evolutionary paths are diverging despite shared genetic targets. Given these nuances, studies seeking to quantify gene reuse should, whenever possible, also assess allele reuse. Several tools and approaches facilitate this assessment. Targeted long‐read sequencing of reused gene regions can identify whether repeated adaptation is driven by synonymous or nonsynonymous changes in causal variants identified through outlier analysis (Konečná et al. 2021; Iyer et al. 2024). Methodologically, a modelling framework, like AF‐VapeR can be used which detects parallel allele frequency shifts and can quantify allele reuse by identifying shared outliers with similar or divergent allele frequency changes across lineages (Whiting, Paris, Parsons, et al. 2022; Whiting, Paris, Van Der Zee, and Fraser 2022; McCulloch et al. 2021; Figure 3B). Lastly, to determine the mechanism of allele reuse, the Distinguishing Modes of Convergence (DMC) framework (Lee and Coop 2017) can differentiate between reuse from standing genetic variation and independent de novo mutations (see Table 1 for summary, also discussed in the next section).

Together, these approaches provide a powerful toolkit for disentangling gene reuse from allele reuse, revealing whether repeated adaptation reflects shared evolutionary histories at the genic or allelic levels, or both.

## 6 Identifying the Mechanisms of Gene and Allele Reuse

To address the challenge of discerning the mechanisms underlying repeated adaptation, particularly in scenarios involving complex evolutionary histories, model‐based statistical frameworks that leverage population genomic data can be useful. Using these models, one can primarily ask two questions: (a) how many genes or alleles are reused across lineages during repeated adaptation? and (b) how many of these reused genes or alleles arose through the same underlying mechanism of adaptation?

When positive selection acts on a beneficial allele, it can reduce diversity at nearby neutral sites through genetic hitchhiking (Smith and Haigh 1974; Stephan et al. 2006). This process causes nearby neutral alleles to deviate more strongly from their original frequencies—some rising much higher and others being lost—resulting in greater variability in allele frequencies than expected under neutrality (Lee and Coop 2017). Building on this framework, Lee and Coop (2017) extended coalescent theory to examine how shared genetic hitchhiking events between lineages increase the covariance in allele frequencies at a gene near a selected site in the genome. They further expanded this framework to incorporate different models of migration and selection acting on standing genetic variation. Using this theoretical foundation, they developed a statistical approach to infer the evolutionary origins of repeated adaptation by distinguishing among three key modes (as described in the background section): independent or de *novo* mutations, SGV, and migration (Figure 3C). Their model explicitly tests how positive selection influences allele frequency covariance at neutral sites linked to a beneficial allele, depending on the mechanism of adaptation. By incorporating these hitchhiking effects into a model that accounts for population structure, they implemented a composite likelihood method capable of analysing genomic data (mainly a set of SNPs) from multiple lineages to test for repeated adaptation. This approach allows researchers not only to identify genes involved in repeated adaptation but also to estimate parameters such as the strength and timing of selection and the mechanism of origin of reused alleles. The utility of this framework is demonstrated through two empirical applications: *Mimulus guttatus* lineages adapting to copper‐contaminated soils, where repeated adaptation was driven by selection on standing variation (Wright et al. 2024), and *Fundulus heteroclitus* lineages rapidly adapting to pollution, where both independent mutations and gene flow contributed to repeated adaptation (Reid et al. 2016).

With easy implementation, this model can be used to quantify the degree of gene or allele reuse as well as determine the number of reused genes or alleles that originate from each of the three mechanisms. For example, suppose 80% of the shared SNPs in Step 4 of Figure 2 are genes or alleles shared between two lineages. This model will first estimate the frequency of the best‐fit models that explain the maximum likelihood for each of the three mechanisms for each shared gene. This frequency is then used to identify how many of these genes or alleles come from de novo mutation, SGV, or gene flow, thereby enabling the identification of a specific mechanism (Figure 3D). This approach has been successfully applied to determine the mechanism and extent of gene and allele reuse during repeated adaptation. For example, in *Arabidopsis arenosa* , researchers used this model to identify the mechanisms underlying repeated adaptation to serpentine soil. The model revealed that 97% of the shared SNPs underlying repeated adaptation in this system are present in standing genetic variation in the adaptive lineages (Konečná et al. 2021). Interestingly, a single SNP associated with the *TWO PORE CHANNEL1* (TPC1) gene originated repeatedly through de novo mutation. In a study of molecular adaptation to highlands in maize landraces, researchers used this model to show that most repeatedly selected SNPs originated via multiple mechanisms, including migration from a single population, and standing variation introduced by ancient gene flow (Wang et al. 2021).

Studies have also inferred allelic repeatability during adaptation. Zhang et al. (2019) utilised the Lee and Coop method to demonstrate significant allele reuse in highland adaptation among maize populations. They showed that in all maize lineages included in their study, 97% of the shared outlier SNPs showed frequency shifts in the same allele (Zhang et al. 2019) indicating allele sharing through the same gene, same allele scenario (Figure 3A). These examples reemphasize our point above regarding differentiating between gene reuse versus allele reuse during repeated adaptation. Furthermore, this approach adds another layer to quantifying the degree of gene reuse by explicitly identifying the mechanism underlying the reuse of each gene during repeated adaptation.

## 7 Problems and Solutions to Validate Evidence of Gene Reuse

Detecting gene reuse from genomic data is sensitive to analytical methods, requiring careful cross‐validation before and after running tests. Given the methodological (e.g., insufficient power to detect gene reuse) and biological (e.g., population structure or complex genomic architecture) issues that can complicate the detection of gene reuse, cross‐validation of methods to identify shared genes between lineages is essential to confirm evidence of repeated adaptation. For highly polygenic or omnigenic traits, adaptation may occur without strong allele frequency shifts at multiple small‐effect genes, necessitating impractically large sample sizes to detect subtle but meaningful signals. Additionally, biological factors such as complex genetic architecture and gene interactions can further obscure signals of gene reuse by distributing adaptive changes across many genes. Insufficient genome scan power limits our ability to infer gene reuse, as demonstrated by the significant drop in the probability of detecting selection at the same gene in multiple lineages under imperfect power (Booker et al. 2023). Here we highlight some potential issues that can complicate the detection of gene reuse. We then suggest possible cross‐validation approaches that can help fix these issues.

### 7.1 Issues With Detecting Gene Reuse Correctly

#### 7.1.1 Sample Sizes

Like any population genomics study, insufficient sample sizes per lineage could lead to incorrect estimations of demographic parameters such as effective population size and population structure, which could in turn inflate or deflate perceived LD across the genome, adding uncertainty to the analysis of gene reuse (Subramanian 2016; McLaughlin and Winker 2020). Small sample sizes can also negatively impact the detection of candidate adaptive genes, particularly those with small effects. Additionally, smaller sample sizes may mean that small‐effect genes or rare genes could go undetected during the genome scans, leading to a biased set of genes that are used to identify and quantify gene reuse (Hong and Park 2012). Therefore, it is essential to consider sample sizes before designing a study to measure the extent of gene reuse during repeated adaptation.

#### 7.1.2 Genomic Sequencing and Variant Calling

Sequencing strategies can have a significant impact on detecting adaptive genes and thus quantifying gene reuse. If there is no reference genome for the focal study lineages, calling SNPs and identifying causal genes using short‐read genomic sequencing data can be unreliable, potentially leading to the detection of false positives as candidate adaptive outliers (Valiente‐Mullor et al. 2021). Generally, it is assumed that for short‐read data, the genes detected as adaptive are mostly linked to an adaptive allele (Chaturvedi et al. 2018; Abbasi and Alexandrov 2021), and therefore, detecting the exact causal SNPs can be difficult. Adequate depth of genomic sequences is also crucial in making sure that both small and large effect genes, as well as common and rare variants, are detected when identifying candidate adaptive genes.

The availability of a reference genome can be crucial, particularly when identifying repeated adaptation between diverged (or diverging) lineages. In closely related species, a single reference genome can lead to more reliable detection of adaptive SNPs. In diverging lineages, genome duplications or rearrangements may place homologues (i.e., any genes sharing a common ancestor) in different genomic regions as lineages become more diverged (Booker et al. 2023). Thus, identifying repeated adaptation also requires careful consideration of orthology (i.e., homologous genes present in different species that have diverged through a speciation event) and paralogy (i.e., homologous genes within a species or lineage that have originated from a gene duplication event) among lineages (Koonin 2005). If paralogous genes show adaptive signals in different lineages, this can still represent repeated adaptation. The challenge becomes greater for distantly related species with limited synteny or similar genome segments.

#### 7.1.3 Genetic Architecture

In the case of polygenic adaptation, detecting gene reuse remains difficult for several reasons. Beyond the complications of polygenic trait architectures as described above, other issues can complicate gene reuse detection. First, when many genes contribute to a trait, their individual effects and resulting changes in allele frequencies are minor, making it challenging to distinguish them from random genetic drift or demographic fluctuations (Ehrlich et al. 2020). Second, genetic redundancy means that only a subset of the alleles affecting a trait is needed to reach an optimal state, allowing different populations to adapt using different combinations of genes (Hunter 2022). Third, as the additive effects of individual genes decline, interactions between genes become more important in the long term (Hallander and Waldmann 2007; Paixão and Barton 2016), further reducing the likelihood of shared genetic responses across replicates. These factors, combined with the potential for demographic and background processes to mimic the effects of selection, make it challenging to detect polygenic adaptation in the wild. Given that the effects of individual genes underlying adaptation are normally expected to be small, the genomic signature of a particular genetic marker might not be strong enough to detect selection. As a result, more methods, such as the C‐score method, are needed to investigate the genetics of adaptation at polygenic traits.

#### 7.1.4 Genome Scans

Genome scan approaches are used extensively to identify candidate genes underlying adaptation. However, it is essential to note that signals resembling repeated adaptation can arise from other processes that produce extreme test statistics in orthologous genomic regions. Linked selection, such as genetic hitchhiking, may cause a nearby neutral gene to appear adaptive if synteny is conserved across lineages (Lee and Coop 2017; Booker et al. 2023). Similarly, background selection in regions of high functional density and low recombination can produce false positives, mainly if recombination landscapes are conserved (Chaturvedi et al. 2018, 2022). Due to this confounding effect, multiple adjacent windows can show significant signals linked to a gene with known major effects. Therefore, genome scans for repeated adaptation should be considered an initial screening step, requiring further validation to rule out confounding processes.

Along these lines, GEA methods are now extensively used for reverse genetics studies to identify adaptive genes using a genome scan approach. However, these methods come with their own limitations. For instance, GEA methods, such as Redundancy Analysis (RDA) (Forester et al. 2016, 2018), Latent Factor Mixed Models (LFMM) (Frichot et al. 2013), Weighted‐Z method (WZA) (Booker et al. 2024), and BayPass (Gautier 2015), can be affected by sample sizes and environmental variability when detecting outlier genes under selection (Booker et al. 2024). Particularly, LFMM and BayPass can yield different sets of candidate loci from the same data set. Studies show LFMM often identifies more environment‐associated SNPs because it uses less stringent population structure corrections whereas BayPass provides a more conservative set of candidates (Booker et al. 2024). GEA methods that do not account for population structure exhibit higher power to detect local adaptations and lead to false signals of local adaptation (e.g., Lotterhos 2019; Salmón et al. 2021; Hartke et al. 2021). A cross‐validation approach that estimates candidate genes using multiple methods and then utilises only the common gene set for quantifying repeated adaptation is often recommended. We detail these solutions below.

### 7.2 Solutions for Correctly Identifying Gene Reuse

Addressing both statistical and biological challenges is essential for accurately interpreting patterns of gene reuse in adaptive evolution. While not all methodological issues can be fully resolved, here we suggest strategies to validate results and minimise false positives in these studies.

A solid study design that includes appropriate sample sizes based on the study goals and analyses, along with a sequencing strategy that considers depth of coverage, can reduce genotyping errors and increase the likelihood of detecting a robust set of candidate genes and generating reliable SNP‐based allele frequencies. For diploid non‐model organisms, recommended sequencing depths vary by approach: typically > 20× coverage per individual for RAD sequencing, ~1–4× coverage for low‐coverage whole‐genome resequencing, and > 50–100× coverage per pool for pooled samples analysed using Pool‐Seq (Schlötterer et al. 2014; Fuentes‐Pardo and Ruzzante 2017; Watowich et al. 2025). Since different variant calling programs or settings can influence results, it is advisable to apply strict filtering criteria to the identified SNPs, ensuring only the most reliable sites are used in subsequent analyses (also see Hoban et al. 2016).

A window‐based genome scan approach can be a more appropriate method for identifying candidate adaptive genes and estimating gene reuse across multiple lineages. This is because complex genetic architectures and LD can obscure the identification of adaptive genes, and a window‐based approach can help avoid misleading signals caused by linkage disequilibrium (Booker et al. 2023). The choice of window size is critical: if windows are too narrow relative to local linkage disequilibrium, multiple windows may correspond to the same gene, requiring stringent multiple testing corrections, while overly broad windows risk diluting selection signals by including linked neutral sites. Ultimately, window size should be chosen based on recombination rates, linkage decay, and the need to balance resolution with statistical power (Beissinger et al. 2015; Booker et al. 2020). When comparing multiple lineages to a single reference genome, the challenge of orthologs and paralogs in diverging lineages can be fixed by using tools such as OrthoFinder (Emms and Kelly 2019), which groups genes into orthogroups—sets of genes descended from a single ancestral copy—thereby allowing comparisons of adaptation at the orthogroup rather than the single‐gene level (Yeaman et al. 2016, 2018; Booker et al. 2023).

To avoid potential issues associated with different GEA methods, multiple genome scan approaches can be cross‐validated to determine whether each method detects the same selection outliers (Figure 2, Step 1). Overlap methods can then confirm if the same genes are identified as outliers by all methods, and only those genes should be retained for gene reuse analysis. After identifying the candidate genes, triangulation can verify if the correct genomic region has been pinpointed as a shared outlier. Alternatively, randomization tests, such as those used by Chaturvedi et al. (2022), can identify false results from GEA methods: they randomised climate variables during BayPass runs, thereby ruling out significant shared genes in pairwise species comparisons but detecting significant results across more than two species (Chaturvedi et al. 2022). This indicates that different validation criteria can highlight the importance of integrating multiple approaches to ensure robust conclusions. Like the GEA methods, multiple significance tests can be used to cross‐validate whether the gene set overlaps detected between lineages are significant. The set of genes that are detected as reused or significantly shared according to multiple methods is more likely to be correctly identified as drivers of repeated adaptation.

## 8 Conclusion

Detecting gene reuse during repeated adaptation remains a formidable challenge, shaped by both methodological constraints and the inherent complexity of biological systems. Many existing approaches rely on identifying shared genes across independently adapted populations, yet factors such as background population structure, genetic architecture of traits, and gene–gene interactions can obscure signals of gene reuse. Our review underscores the need for more precise identification methods that account for these complexities. We highlight recent methodological advancements that better integrate genetic architecture and emphasise the importance of distinguishing between gene reuse and allele reuse. Additionally, we advocate for incorporating models that identify the mechanism of gene reuse. Finally, we call for rigorous cross‐validation of statistical tests to mitigate false positives arising from sequencing errors or biological confounders. As research in this field advances, continued methodological refinement will enhance our ability to identify gene reuse and quantify its extent with greater accuracy, ultimately leading to more robust insights into repeated adaptation and the predictability of evolution.

## Author Contributions

S.C. conceptualised the project. L.V.D. searched for literature and created the final dataset of published studies to identify methods. L.V.D. and G.S. summarised information from papers to create Table 1. S.C. prepared the manuscript with input from L.V.D. and G.S. All authors reviewed and provided comments on the manuscript.

## Conflicts of Interest

The authors declare no conflicts of interest.

## Data Availability

The data that support the findings of this study are available from the corresponding author upon reasonable request.
